# Chigger mite (Acariformes: Trombiculidae) infestation in reed passerine birds in Central Europe: a case of the bearded tit *Panurus biarmicus*

**DOI:** 10.1017/S0031182022001731

**Published:** 2023-02

**Authors:** Alfréd Trnka, Peter Samaš, Joanna Mąkol

**Affiliations:** 1 Department of Biology, University of Trnava, Priemyselná 4, Trnava 91843, Slovakia; 2 Institute of Vertebrate Biology, Czech Academy of Sciences, Květná 8, Brno 60365, Czech Republic; 3 Department of Invertebrate Systematics and Ecology, Wroclaw University of Environmental and Life Sciences, Kożuchowska 5b, Wroclaw 51-631, Poland

**Keywords:** Avian host, barcoding, morphology, parasite, Parasitengona, prevalence

## Abstract

Larval trombiculid (chigger) mites are common ectoparasites of terrestrial vertebrates including humans, causing itching and skin inflammation known as trombiculiasis. Investigation of their diversity, distribution and seasonal abundance is therefore important from a veterinary and public health point of view. Although researchers have paid increased attention to these parasites in recent years, there is still little ecological data available on chiggers associated with birds inhabiting different types of habitats such as wetlands, for example. In 2021, we investigated the mite fauna in a specialist reedbed passerine, the bearded tit (*Panurus biarmicus*), and their effects on this host in the south-west Slovakia, Central Europe. A total of 1134 larvae of 1 mite species *Blankaartia acuscutellaris* were found in 99 out of 267 examined bearded tits. Juveniles were more infested than adult birds, but no differences were found between sexes. The larvae of mites first appeared on the host during the second half of June and peaked in the second half of July. After that, their numbers decreased gradually until October. Despite the relatively high prevalence and intensity of mite infestation in the bearded tit, no differences in body condition between infested and uninfested birds suggest that infestation by *B. acuscutellaris* may not have serious negative effects on the host health. Bearded tits can therefore be a reliable indicator of the presence of the chigger mites in wetland habitats.

## Graphical abstract

**Figure:**
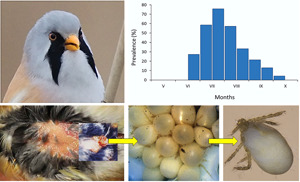

## Introduction

Birds harbour a high diversity of ectoparasites, including mites (Acariformes). While some of these parasites are relatively benign, others can have important detrimental consequences for host fitness and health (Proctor and Owens, 2000; Stock, 2019). Mites that feed on skin, for example, can induce cutaneous inflammation, anaemia and even death caused by starvation and exhaustion (Literák *et al*., 2001; Murillo *et al*., 2020). Moreover, mite infestation may indirectly affect susceptibility to other diseases, predation and behaviour of parasitized birds. However, while pathogenic effects of mites are well known for domestic and captive birds, the distribution and abundance of these ectoparasites and their impact on wild bird populations is poorly studied (Atkinson *et al*., 2008; Muñoz *et al*., 2022).

One of the most common mites that infect birds are chigger mites from the family Trombiculidae s.l. (=*sensu* Kudryashova, 1998). There are more than 3000 species of trombiculid mites parasitizing a wide range of hosts including domestic and wild birds, mammals, lizards, amphibians as well as humans (Nielsen *et al*., 2021). Most of them are considered generalists which colonize any available host species present in their preferred habitats; others favour certain animal classes such as birds, or specialize on a particular species (O'Callaghan *et al*., 1994). Only the 6-legged larvae (chiggers) are parasitic, while nymphs and adults are free-living predators that dwell in the soil and litter and feed on other arthropods and their eggs. After hatching, larvae of chigger mites wait for a suitable host or display a directed short-distance movement towards the host (Sasa *et al*., 1957; Wohltmann, 2000). Once a host is found, they attach to the surface of its skin and begin to release salivary secretion. Lytic enzymes in saliva allow them to suck macerated host tissue (Literák *et al*., 2001). Larvae parasitize for 2–12 days, however, the prolonged contact with the host, going beyond the parasitic phase can also occur (Clayton and Walther, 1997; Moniuszko and Mąkol, 2016). At that time, they are easily identified as tiny orange or red immobile dots usually clustered in unfeathered areas of the skin.

Trombiculid mites live in a variety of habitats, mainly in forests and grasslands, but also in swamps. Despite the increasing number of papers published in the recent years (e.g. Literák *et al*., 2001, 2007*a*, 2007*b*, 2008; Chau, 2005; Dietsch, 2005; Stekolnikov *et al*., 2007, 2022*a*; Mąkol and Korniluk, 2017; Bassini-Silva *et al*., 2018; Kalúz *et al*., 2018), bird-associated Trombiculidae have been studied to a much lesser extent in comparison with chiggers that infest mammals. Moreover, the prevalence of their larvae in Central Europe has been studied mainly in birds inhabiting terrestrial forest and steppe habitats (Daniel, 1961; Literák *et al*., 2001, 2007*b*), while birds occupying wetlands were largely overlooked by researchers.

This study investigated the chigger mite fauna in a small passerine, the bearded tit (*Panurus biarmicus*), inhabiting large reedbeds in extensive flooded marshes (Robson, 2007). Given that chigger mites can be responsible for, among others, restricted growth and loss of weights in birds (Literák *et al*., 2001), and low weight associated with weakened health can contribute to marked fluctuations of bearded tit populations over time (Surmacki and Stępniewski, 2007; Stępniewski and Halupka, 2018), relationship between the sex, age and body condition of investigated birds, and the prevalence and load of mite larvae were also examined. We predict that increased mite loads lead to a decreased body condition in infected birds.

## Materials and methods

### Study species and study site

The bearded tit is a relatively small (14–15.5 cm in length and 11–21 g in weight), socially monogamous passerine with a strong sexual dimorphism and biparental care (Cramp and Perrins, 1993). As a wetland specialist, it breeds in large beds of *Phragmites* reed and associated dense tall vegetation by or in water. Pairs are formed in juvenile flocks and remain together for life (Griggio and Hoi, 2011). They may produce 2–5 broods per season (Stępniewski and Halupka, 2018). Bearded tits are resident or migrate a short distance only. During the non-breeding season, they live in flocks (Cramp and Perrins, 1993).

The field study was carried out in the Parížske močiare marsh located near the villages of Gbelce and Nová Vieska (47°51N, 18°29E) in the south-west Slovakia. The marsh represents one of the largest wetlands in Slovakia. It covers an area of 184 ha and comprises extensive reedbeds of *Phragmites australis*, with fringing areas of other aquatic plants. The size of the breeding population of bearded tits was estimated to be between 25 and 50 breeding pairs in this locality (Trnka *et al*., 2003).

### Data sampling

The samples were collected once every 1–2 weeks between 9 May and 5 October 2021 (a total of 23 sampling days). The bearded tits were caught in mist-nets placed in the reedbeds. A total of 267 birds were examined, 37 of which were adults (21 males and 16 females), 135 juveniles (78 males and 57 females) and 95 of unknown age (44 males and 51 females). Individuals that were recaptured within the next 14 days were not included in this analysis as they could still be harbouring mites detected in the previous inspection.

Each captured bird was ringed, sexed, aged (if possible) as first calendar year bird or older (adult), measured and then visually examined for the presence of mite chiggers by the naked eye. The body mass was weighed with a Pesola spring balance to the nearest 0.1 g and the minimum length of the tarsus was measured with a sliding calliper to the nearest 0.1 mm.

Visual inspection of birds included focal areas where chigger mites regularly colonize, in particular the ventral part of the body between the sternum and the cloaca and behind the legs and wings. When a bird was parasitized, the exact number, counted using a hand glass, and location of mite larvae were recorded and part of their colonies carefully removed using tweezers and preserved in 70% alcohol. Captured birds were then released back into the wild. No bird died or was injured during this procedure.

### Mite identification

One to 2 mite larvae randomly selected from 81 hosts were intended for morphological and molecular examination. For molecular analysis a non-destructive method of DNA extraction aimed at retaining the exoskeletons for morphological examination was used. Total genomic DNA was extracted from non-pooled samples (single mites taken from different host specimens) using a DNeasy Blood and Tissue Kit (Qiagen, Hilden, Germany). The mites were transferred from 96% ethanol to 180 *μ*L ATL lysis buffer with 20 *μ*L proteinase K. The samples were incubated for 3 days at 56°C with a mixing speed of 350 rpm. After digestion, the lysis buffer containing nucleic acids was transferred to a new Eppendorf tube and subjected to DNA isolation according to the manufacturer's protocol. Amplification of the cytochrome c oxidase subunit 1 (COI) fragment was carried out using degenerate forward primer bcdF01 (5′-CATTTTCHACTAAYCATAARGATATTGG-3′) and reverse primer bcdR04 (5′-TATAAACYTCDGGATGNCCAAAAAA-3′) (Dabert *et al*., 2010) under the following thermocycling conditions: 95°C/3 min – initial denaturation; 95°C/30 s – denaturation, 50°C/30 s – annealing, 72°C/45 s – extension – 40 cycles; 72°C/7 min – final extending. The polymerase chain reaction (PCR) (25 *μ*L) was carried out using the following PCR mix: 5 *μ*L of genomic DNA, 3 *μ*L of each primer, 1.5 *μ*L of water and 12.5 *μ*L of KAPA2G Robust HotStart ReadyMix (Merck KGaA, Darmstadt, Germany). The amplification product was purified using a QIAquick PCR purification kit (Qiagen, Hilden, Germany) and sequenced in both directions (Genomed S.A., Warsaw, Poland). The multiple sequence alignment was performed in Geneious 9.1.8 (https://www.geneious.com). The non-homogeneous sequences obtained in this study are deposited in the GenBank under the accession numbers: OP945735, OP945736 and OP945737. For the purpose of comparison, 10 COI sequences (accession nos. KY930734.1, KY930735.1, KY930736.1, KY930737.1, KY930738.1, KY930739.1, KY930740.1, KY930741.1, KY930742.1 and KY930743.1) of *Blankaartia acuscutellaris* were retrieved from the GenBank. Distance calculation between sequences was performed in Geneious 9.1.8 with default parameters.

**Fig. 1.**
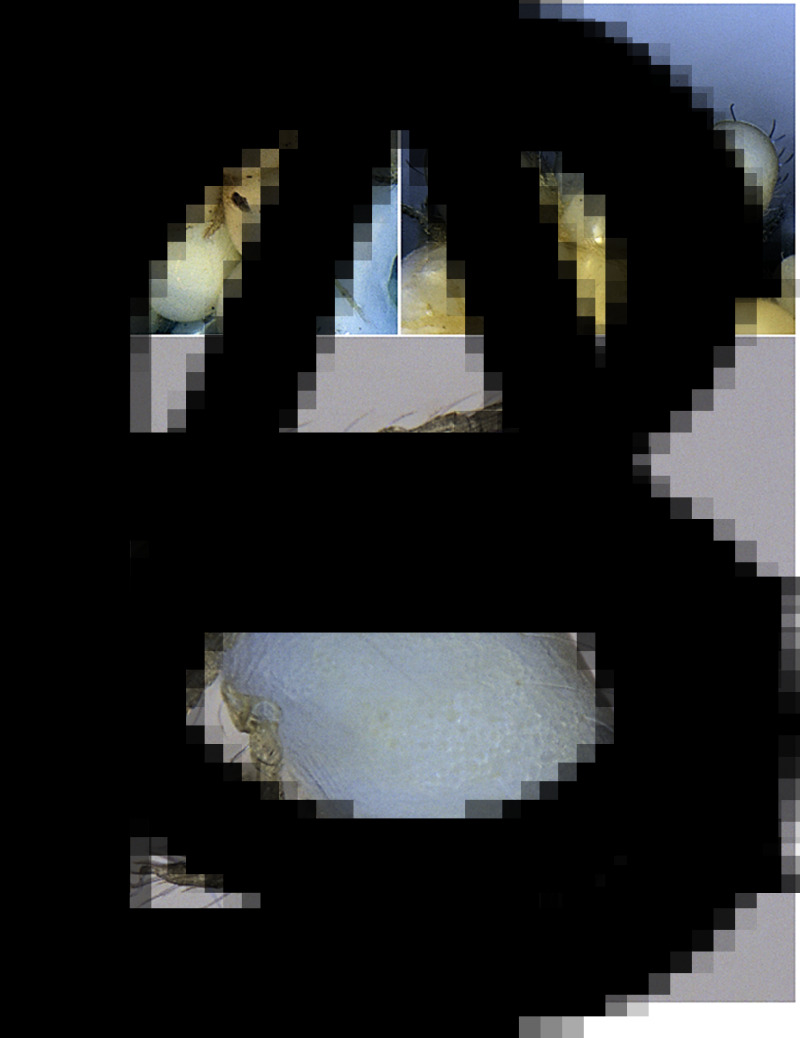
*Blankaartia acuscutellaris* collected from *Panurus biarmicus*: (A) a cluster of larvae; (B) larvae at different levels of engorgement and (C) larva separated from the cluster. Not to scale.

**Fig. 2.**
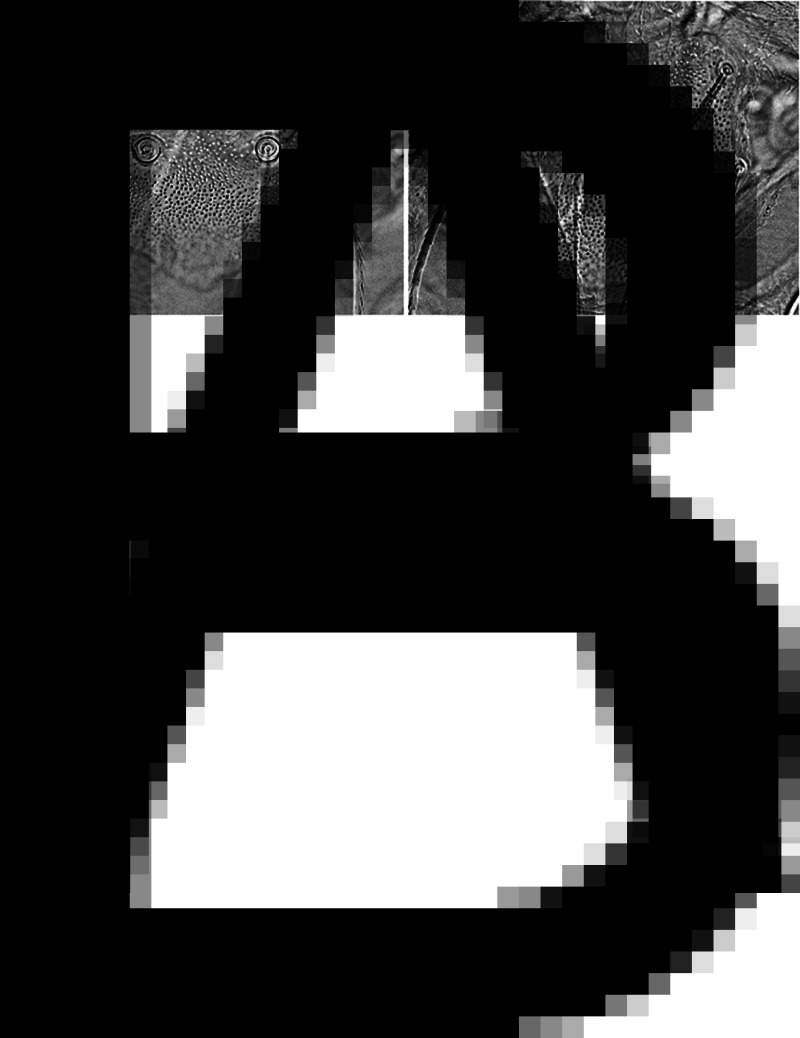
Variation in the shape of scutum in larvae of *B. acuscutellaris* collected from *P. biarmicus*: (A) scutum with postero-lateral margins forming an angle and (B) scutum rounded posteriorly.

The exoskeletons remaining after DNA extraction were mounted on microscopic slides in Berlese's gum chloral fluid (TCS Biosciences Ltd, Buckingham, United Kingdom). Morphological analyses were carried out in a Nikon Eclipse 80i compound microscope, equipped with differential interference contrast and DS-Fi3 camera system, using NIS-Elements D software (https://www.microscope.healthcare.nikon.com/products/software/nis-elements/nis-elements-documentation). Chigger terminology follows Goff *et al*. (1982). To identify larvae to genus and species levels we referred to Kudryashova (1998), Fernandes and Kulkarni (2003) and Stekolnikov (2018). Photographs of alcohol-preserved larvae (Fig. 1) were shot with Imaging Source, 5 MP camera combined with a Nikon SMZ25 stereomicroscope; microphotographs of scutum in slide-mounted larvae (Fig. 2A and B) – with the DS-Fi3 camera system combined with Nikon Eclipse 80i. The measurements of morphological structures in larvae are given in micrometres.

The mites used for morphological analyses, including exoskeletons retained after DNA extraction, are deposited in the acarological collection of the Department of Invertebrate Systematics and Ecology, Wrocław University of Environmental and Life Sciences, Poland.

### Statistical analysis

A univariate linear model was used to test effects of infestation status (binary; infested or not), date (continuous; Julian date of catching and measuring an individual) and its second-degree polynomial, and sex (binary; male or female) on response variable body condition (continuous; linear regression residuals of body weight on tarsus length). Interaction effects of infestation status with age, infestation status with sex and infestation status with date as a second-degree polynomial were also added. Next, we ran second linear model with the same model structure but we replaced the effect of infestation status with infestation intensity (integers; number of parasite individuals). To interpret main coefficients involved in the interactions or polynomials, all the independent variables entered the model were centred (subtracted the sample mean from all the variable values) (Schielzeth, 2010). Marginal means estimated by a linear model were calculated using the R package emmeans v. 1.7.5 (Lenth, 2022). Outputs of full models were always presented. Potential collinearity among the covariates was low, variance inflation factors were <1.5 in all cases (Zuur *et al*., 2010). Model residual diagnostics was performed with R package DHARMa v. 0.3.3.0 (Hartig, 2022). Statistical analyses were conducted in R v. 4.1.3 (R Core Team, 2020).

## Results

### Mite prevalence and abundance

**Fig. 3.**
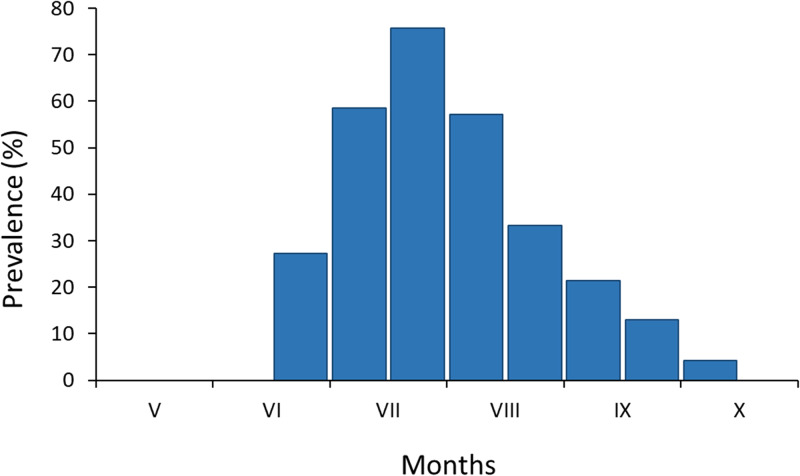
Seasonal prevalence of *B. acuscutellaris* larvae on *P. biarmicus* host.

The larvae of trombiculid mites were found in 99 out of 267 examined bearded tit individuals (37.1%). Juveniles were parasitized more frequently than adults (62.9 *vs* 16.2%, respectively, *χ*^2^ = 25.47, *df* = 1, *P* < 0.001), but no significant differences were found in the prevalence of infestation between the sexes (males *vs* females: 40.6 *vs* 33.1%, *χ*^2^ = 3.59, *df* = 1, *P* = 0.206). The larvae of mites first appeared during the second half of June and then their prevalence increased sharply to reach the peak during the second half of July (Fig. 3). Thereafter, it decreased gradually from August to early October.

A total of 1134 larvae were found on the examined bearded tits, with the average number ± s.d. of 11.46 ± 11.23 specimens per parasitized host (min–max = 1–95). There were no significant differences in the intensity of infestations between juveniles and adults (mean ± s.d. = 12.1 ± 11.6 and 10.5 ± 13.0, respectively; Wilcoxon rank-sum test, *W* = 188, *P* = 0.25), nor between males and females (mean ± s.d. = 11.9 ± 13.2 and 10.8 ± 7.9, respectively; Wilcoxon rank-sum test, W = 1197, P = 0.96). Most parasitized birds (74.7%) had mites grouped in 1 cluster; the presence of 2 clusters was detected in 21.2% of hosts, whereas 3 clusters were present in 4 host specimens only. The mean number of larvae (± s.d.) per cluster was 8.8 ± 7.12 specimens (min–max = 1–61, n = 128 clusters). The preferred sites of attachment of larvae were ventral and lateral parts of the body between the cloaca and sternum (46.1%), below wings (27.4%) and in close proximity to the cloaca (19.5%). Single clusters of mites were also rarely observed on the breasts.

### Mite identification

Of 81 samples (=bird specimens) designed for molecular analyses we obtained 52 COI sequences from larvae collected from different host specimens (for the remaining samples the analyses failed). The barcoding success equalled 64%. The sequence alignment and cutting resulted in a compact 598 bp data block. Forty-eight sequences were identical, and 4 others differed by 1–2 nucleotide substitutions. The percentage of identical bases varied between 99.7 and 100. Due to the high level of identity of sequences, we could confirm the common specific affiliation of the examined mites. A comparison of our sequences with those retrieved from the GenBank and obtained from larvae collected from rodents *Bandicota indica* and *Rattus sakeratensis* in Laos (Kumlert *et al*., 2018) revealed an 83.3–84.6% identity.

The morphological analyses resulted in ascertaining the following character states in the examined larvae: gnathobase with cuticular puncta arranged in transverse lines; galeala nude; cheliceral blade with tricuspid cap; palpfemorala, palpgenuala – with setules; odontus with 3 prongs, divided at c. half length; 2 nude setae and 1 setulated seta on palpal tibia; palpal tarsus with 7 setulated setae, 1 smooth subterminal seta (subterminala) and 1 solenidion; scutum pentagonal in outline, punctated; eyes present, each composed of 2 lenses; 2 humeral setae and 24–30 setae arranged in horizontal rows on opisthosoma dorsum; 10–14 ventral setae present posterior to legs III; total number of setae on opisthosoma: 40–44; coxae punctated with ±linear layout of puncta; 1 seta present on each coxal plate; 1 pair of setae present between coxae II and 1 pair – between coxae III; genu I with 4 normal setae and 3 solenidia, genu II and III – each with 3 normal setae and 1 solenidion; solenidion on tarsus I large, about 20 *μ*m in length; companion seta (companala) on tarsus I smooth; mastitarsala III present, all tarsi terminated with 2 claws and claw-like empodium. The morphometric data are provided in Table 1.

**Table 1.** Metric data for *Blankaartia sinnamaryi* and *Blankaartia acuscutellaris* [partly after Mąkol and Korniluk (2017) and Stekolnikov *et al*. (2022*a*)]

	*B. sinnamaryi*		*B. acuscutellaris*^a^	
	[Typical form] Stekolnikov *et al*. (2022*a*) (*n* = 14)	[Large form] Stekolnikov *et al*. (2022*a*) (*n* = 3)	Mąkol and Korniluk (2017) (*n* = 8)	Present study (*n* = 10)
AW	73–83	88–91	66–73	80–87
PW	77–86	96–99	68–77	86–94
SB	35–45	44–45	27–30	39–43
ASB	23–31	29–32	24–32	28–33
PSB	40–50	47–47	39–45	43–50
SD	62–77	76–79	65–76	72–84
P-PL	27–36	31–32	29–34	31–38
AP	21–32	33–34	26–31	30–33
AM	33–50	56–63	44–52	63–70
AL	30–39	43–46	30–37	38–44
PL	54–66	79–81	61–69	78–93
S	50–65	80–85	50–62	79–93
DSmax	54–63	72–77	63–80	73–84
VSmax	46–57	60–72	–	52–71
Cx I	–	–	48–64	53–66
Tr I	–	–	22–36	27–41
bFe I	–	–	27–38	29–40
tFe I	–	–	26–33	30–42
Ge I	–	–	38–45	37–44
Ti I	–	–	45–51	47–56
Ta I	–	–	63–79	84–91
Leg I	346–387	445–450	300–322	326–367
Cx II	–	–	58–64	62–69
Tr II	–	–	26–33	27–43
bFe II	–	–	22–30	32–38
tFe II	–	–	21–32	27–31
Ge II	–	–	27–32	31–35
Ti II	–	–	39–50	45–52
Ta II	–	–	57–68	71–82
Leg II	313–346	364–382	267–294	309–334
Cx III	–	–	56–70	62–72
Tr III	–	–	36–43	45–52
bFe III	–	–	29–36	38–44
tFe III	–	–	23–33	28–33
Ge III	–	–	31–38	37–46
Ti III	–	–	50–58	59–70
Ta III	–	–	76–84	95–100
Leg III	360–396	427–446	310–353	381–408
IP	1024–1129	1255–1258	878–944	1027–1090

Abbreviations (refer to a particular structure or its length): AL, anterolateral, non-sensillary seta on scutum; AM, anteromedian seta on scutum; AP, distance between the bases of AL and PL, on 1 side of symmetry axis; ASB, distance between the anterior margin of scutum and the level of sensilla (S); AW, distance between the bases of anterolateral, non-sensillary setae (AL) on scutum; bFe I, II, III, length of basifemur; Cx I, II, III, length of coxa; DSmax, length of the longest dorsal opisthosomal setae; Ge I, II, III, length of genu; IP, *index pedibus*, total length of leg I, II and III, including coxae; Leg I, II, III, total length of leg, including coxa; SD, length of prodorsal sclerite, scutum (SD = PSB + ASB); P-PL, distance between the level of posterolateral setae (PL) and the extreme posterior margin of scutum; PL, posterolateral, non-sensillary seta on scutum; PSB, distance between the level of sensilla and the posterior margin of scutum; PW, distance between the bases of posterolateral, non-sensillary setae (PL) on scutum; S, sensillum on scutum; SB, distance between the bases of sensilla (S); Ta I, II, III, length of tarsus; tFe I, II, II (L), length of telofemur; Ti I, II, III, length of tibia; Tr I, II, III, length of trochanter; VSmax, length of the longest ventral opisthosomal setae.

a For comparison of metric data on *B. acuscutellaris* after Womersley (1948), Kudryashova (1983) and Ripka and Stekolnikov (2006), see Mąkol and Korniluk (2017).

### Mite infestation and host body condition

**Fig. 4.**
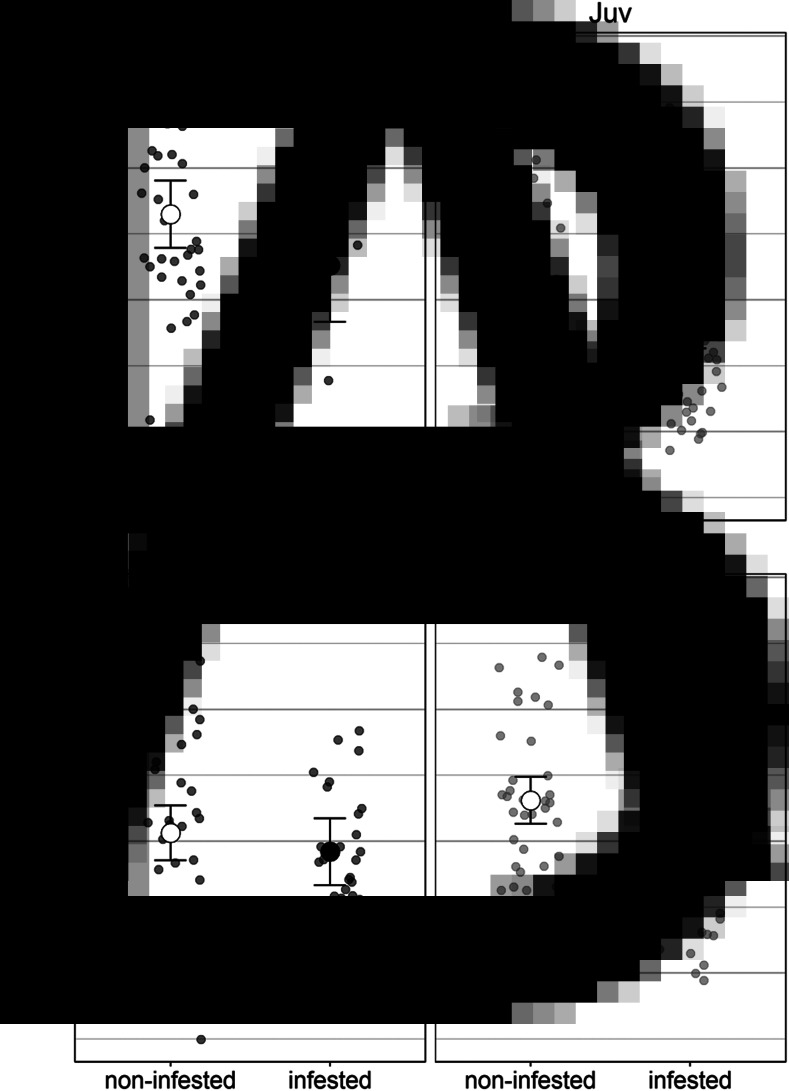
Comparison of body condition indices of infested and non-infested bearded tits according to their age (A) and sex (B). Raw values (grey points) with model estimated marginal means (white and black circles) and their 95% confidence intervals are plotted.

**Fig. 5.**
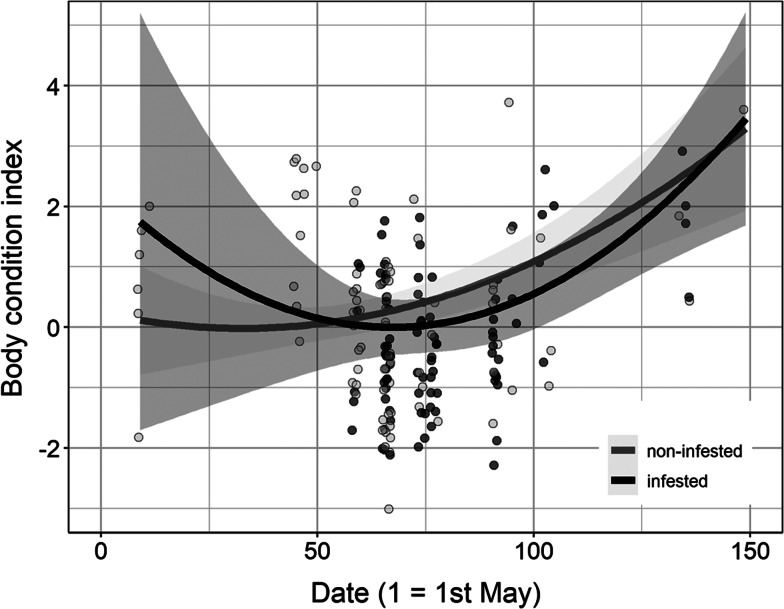
Body condition of infested and non-infested bearded tits during the study period. Grey points represent raw values and lines with their 95% confidence bands were predicted from linear model.

Body condition did not differ between infested and uninfested bearded tits (Table 2). On the other hand, adult birds had greater body condition than juveniles for both non-infested (estimate ± s.e. = 1.85 ± 0.30, *F*_1,162_ = 39.00, *P* < 0.01) and infested bird individuals (1.02 ± 0.45, *F*_1,162_ = 5.09, *P* = 0.03; Fig. 4A). Males had greater body condition than females but only in non-infested bird individuals (0.50 ± 0.24, *F*_1,162_ = 4.27, *P* = 0.04; Fig. 4B). Body condition also changed non-linearly with advancing season in both non-infested (*t*_162_ = 2.53, *P* = 0.01) and infested bird individuals (*t*_162_ = 1.98, *P* = 0.049), being higher in the start and end of the season (Fig. 5).

**Table 2.** Linear model output testing effect of infestation (binary; infested or not), date in season, sex and age category on body condition (expressed as residuals from linear regression of mass on tarsus length)

Parameter	Estimate	s.e.	*df*	*F*	*P*
Intercept	0.00	0.11	1	0.00	0.97
Infested	0.00	0.21	1	0.00	0.99
Date	4.26	1.82	1	–	–
Date^2^	5.36	2.01	2	18.98	<0.01
Sex	0.42	0.17	1	6.32	0.01
Age	−1.41	0.28	1	26.00	<0.01
Infestation:Sex	−0.14	0.33	1	0.18	0.67
Infestation:Age	0.83	0.54	1	2.38	0.12
Infestation:Date	−3.53	3.52	1	–	–
Infestation:Date^2^	3.76	3.84	2	0.60	0.55

Denominator degrees of freedom were 162.

Body condition also changed non-linearly with advancing season in both non-infested (*t*_162_ = 2.53, *P* = 0.01) and infested bird individuals (*t*_162_ = 1.98, *P* = 0.049), being higher in the start and end of the season (Fig. 5). Similarly, we did not detect a significant effect of intensity of infestation on the body condition of the examined birds (Table 3).

**Table 3.** Linear model output testing effect of infestation intensity (number of mite individuals), date in season, sex and age on body condition (expressed as residuals from linear regression of mass on tarsus length)

Parameter	Estimate	s.e.	*df*	*F*	*P*
Intercept	−0.05	0.14	1	0.14	0.71
Infested	0.00	0.01	1	0.02	0.90
Date	4.13	1.49	1	–	–
Date^2^	2.08	1.38	2	4.09	0.02
Sex	0.44	0.26	1	2.78	0.10
Age	−1.14	0.47	1	5.79	0.02
Infestation:Sex	−0.02	0.03	1	0.59	0.44
Infestation:Age	0.04	0.04	1	0.88	0.35
Infestation:Date	0.02	0.15	1	–	–
Infestation:Date^2^	0.00	0.14	2	0.01	0.99

Denominator degrees of freedom were 81.

Six parasitized bearded tits were recaptured at the same site 76–93 days after initial capture. All recaptured birds had increased their body mass by 6.7–26.9% between the first and second captures.

## Discussion

Bearded tits showed relatively high prevalence of mite infestation in the study area, higher than the prevalence found in many other bird species in Central Europe. Of the 14 parasitized species detected by Literák *et al*. (2001) in the Slovak and Polish Carpathians, only the song thrush (Turdus philomelos), the dunnock (*Prunella modularis*) and the Eurasian wren (*Troglodytes troglodytes*) had a greater prevalence (39–50%) than did the bearded tit. The reason for this may be specific foraging strategy of the study species. Soon after independence, while adult birds are still continuing their breeding activity, juveniles form mixed-sex flocks consisting of up to 50 individuals (A. Trnka, personal observation) and feed communally on reeds and margins of open water (Cramp and Perrins, 1993), but very often also on the muddy ground in drier areas of reed-beds, as well as outside reedbeds in the dykes of canals or ponds and elsewhere (Bibby, 1981; van den Elzen, 1993). On the ground, they hop and run quickly and occasionally scratch the ground with their feet like a hen (Cramp and Perrins, 1993). Thus, given that the prevalence and intensity of infestation with trombiculid mites depend significantly on the length of host exposure in an environment with mite occurrence (Literák *et al*., 2001), such feeding behaviour of juvenile bearded tits may favour the high infestation rates by trombiculid mites in this species. This may also explain why juveniles were more infested than adult bearded tits, but no differences were observed between sexes.

The seasonal dynamics of infestation of the bearded tit related probably to dynamics of the mite larvae in the study area. The phenomenon seems to be associated with a long-term evolutionary relationship between the parasite and the host, having its consequences in different phenology of mites which parasitize different groups of hosts. Literák *et al*. (2007*b*) recorded low, 1% prevalence of *Ascoschoengastia latyshevi* on birds during winter months, between December and March in Czech Republic. Zajkowska and Mąkol (2022) observed the presence of *Leptotrombidium* spp. larvae on bats in Poland between July and April of the following year, with the highest numbers recorded in autumn, during bat swarming. Trombiculid larvae associated with rodents were most abundant also in autumn, with special reference to the period from August to October in Central Europe (Daniel, 1958; Moniuszko and Mąkol, 2016; but see Stekolnikov and Mumcuoglu, 2021; Stekolnikov *et al*., 2022*b*). In lowland humid forests, Kalúz *et al*. (1996) found that larvae of *Neotrombicula* species appeared on rodents in the second half of July and peaked in the second half of August, which still seems slightly later than in our study. However, seasonal variation of infestation of hosts' group by mite larvae can be species-specific and influenced by many other factors such as the type of habitat and climatic conditions.

All mites collected from bearded tits in the study area were identified as *B. acuscutellaris*, despite some differences in metric character values (range) between the species studied by Mąkol and Korniluk (2017) based on the material collected in Poland and specimens collected from the bearded tit in Slovakia (Table 1). The relatively high value of the genetic distance between the material examined during the present study and *B. acuscutellaris sensu* Kumlert *et al*. (2018) may question the common specific affiliation of both. However, in the lack of clear morphological differences in qualitative traits, and in the lack of clear metric differences [refer to Fernandes and Kulkarni (2003) in Kumlert *et al*. (2018)], we consider the material conspecific with *B. acuscutellaris*. Future studies will be carried out to elucidate the problem of potential cryptic species within *B. acuscutellaris* complex. Unless more evidence is available, we attribute these differences to geographic but possibly also host-driven variation. The latter phenomenon was discussed by Moniuszko *et al*. (2015) in relations to rodent-associated chiggers. Stekolnikov *et al*. (2022*a*) observed the presence of the ‘typical form’ and the ‘large form’ of *Blankaartia sinnamaryi* in the Neotropics (see also Table 1).

The problem of variation may be facilitated by the relatively wide host spectrum of chiggers but also, and indirectly, by the geographic area covered by the host. The bearded tit is widespread in Europe, Asia and North Africa. In Europe it has a patchy distribution and occurs as 2 subspecies (the third subspecies *Panurus b. kossvigi* is considered extinct, Snow and Perrins, 1998). The nominate subspecies *P. b. biarmicus* is distributed in Western Europe to Sweden, Poland, Italy, Balkans and Transcaucasia, the subspecies *Panurus b. russicus* occurs in Central Europe from Austria, Czech Republic, Slovakia to Balkans and southern Russia (Cramp and Perrins, 1993). Analysis of ringing recoveries from Czech Republic and Slovakia shows that these 2 subspecies may come into contact in the non-breeding period in Central Europe (Hořák *et al*., 2003). Although the species is largely sedentary within its European range, one of the features of bearded tit ecology is regular autumn dispersal movements and irruptions up to a distance of 100–200 km (Hořák *et al*., 2003).

To the best of our knowledge, this is the first report of *B. acuscutellaris* in the bearded tit. This mite species has been previously recorded on another non-passerine bird species [e.g. little bittern (*Ixobrychus minutus*), purple heron (*Ardea purpurea*), ruff (*Philomachus pugnax*), great snipe (*Gallinago media*), Kudryashova, 1998; Mąkol and Korniluk, 2017] that are also closely associated with the reedbeds and other wet places. This corresponds to the habitat rather than host preferences in this mite (Ripka and Stekolnikov, 2006).

Finally, one of the aims of this study was also to find out whether mite infestation has negative impact on the host body condition. Our results failed to confirm this prediction as no significant relationship was found between mite load and body condition of the examined bearded tits. Moreover, body condition of infested and later recaptured birds did not decrease between their first capture and recapture, which can also suggest that infestation by *B. acuscutellaris* has not serious negative effects on the host's health. However, given that many other fitness consequences such as pair bonding, susceptibility to other diseases or to predators etc. (see, e.g. Møller *et al*., 1990), were not studied, the cost of parasitism by this mite species on the bearded tit host needs further investigation.

In conclusion, this study reports for the first time the occurrence of the chigger mite *B. acuscutellaris* on reed-dwelling passerine, the bearded tit, in Central Europe. The relatively high prevalence and intensity of mite infestation in the bearded tit indicates that this species is, in addition to other mite species such as Harpirhynchus dusbabeki (Henry *et al*., 2004; Literák *et al*., 2005; Bochkov and Literák, 2006), a normal host of *B. acuscutellaris*, and that this mite species may be more widely distributed on reed-marsh-dwelling birds than previously thought. In the course of our research, mite larvae were also found in other birds, namely in the moustached warbler (*Acrocephalus melanopogon*), great reed warbler (*Acrocephalus arundinaceus*) and the reed bunting (*Emberiza schoeniclus*) (A. Trnka, unpublished data). Given that larvae of *B. acuscutellaris* have also been reported to attack humans causing trombiculiasis (Fernandes and Kulkarni, 2003; Ripka and Stekolnikov, 2006; Stekolnikov *et al*., 2014), results of this study are also important from a medical and veterinary point of view. We therefore hope that this study will motivate other researchers to study the mite fauna of birds associated with wetland and reedbed habitats.
